# Spinal Dorsal Horn Neurons Receiving Descending Input from the Primary Somatosensory Cortex Contribute to Aβ Fiber-Induced Neuropathic Allodynia in Male Rats

**DOI:** 10.3390/cells14231870

**Published:** 2025-11-26

**Authors:** Sho Shinotsuka, Eriko I, Daichi Sueto, Kazuki Fujimori, Ken Yamaura, Makoto Tsuda

**Affiliations:** 1 Department of Molecular and System Pharmacology, Graduate School of Pharmaceutical Sciences, Kyushu University, Fukuoka 812-8582, Japan; shinotuka.sho.433@m.kyushu-u.ac.jp (S.S.); i.eriko.401@s.kyushu-u.ac.jp (E.I.); sueto.daichi.195@s.kyushu-u.ac.jp (D.S.); fujimori.kazuki.514@s.kyushu-u.ac.jp (K.F.); 2 Department of Anesthesiology and Critical Care Medicine, Graduate School of Medical Sciences, Kyushu University, Fukuoka 812-8582, Japan; yamaura.ken.361@m.kyushu-u.ac.jp; 3 Kyushu University Institute for Advanced Study, Fukuoka 819-0395, Japan

**Keywords:** Aβ fiber, optogenetics, neuropathic allodynia, descending pathway, S1 cortex, spinal cord, rat

## Abstract

**Highlights:**

**What are the main findings?**

- Descending neuronal signaling from the primary somatosensory (S1) cortex to the spinal dorsal horn (SDH) directly contributes to Aβ fiber-derived neuropathic allodynia in male rats.
- Superficial SDH neurons receiving direct projections from S1 cortical neurons integrate excitatory inputs from Aβ fibers and inhibitory inputs from SDH interneurons; loss of this inhibition unmasks their excitatory influence on spinal circuits, leading to allodynia.

**What are the implications of the main findings?**

- This study demonstrates that an aberrant brain–spinal cord circuit involving the S1→SDH pathway underlies the pathological conversion of innocuous touch into pain.
- Targeting this corticospinal pathway may represent a promising therapeutic strategy for neuropathic allodynia.

**Abstract:**

Mechanical allodynia is the predominant symptom of neuropathic pain following peripheral nerve injury (PNI) and is characterized by pain evoked by innocuous sensory signals transmitted through low-threshold mechanoreceptive primary afferents, including Aβ fibers. However, the underlying neural mechanisms remain insufficiently understood. Previous studies have suggested that the pathological conversion of tactile input into nociceptive signals involves maladaptive alterations in neural circuits and function within the spinal dorsal horn (SDH). Somatosensory processing and transmission in the SDH are regulated not only by local neuronal circuits but also by descending inputs from the brainstem and higher cortical regions. In this study, we show that chemogenetic silencing of descending neurons projecting directly from the primary somatosensory (S1) cortex to the SDH (S1^→SDH^ neurons) suppresses both PNI-induced allodynia-like behavior and c-FOS expression in the superficial SDH observed in male rats where touch-sensing Aβ fibers were optogenetically activated. S1^→SDH^ neurons were excitatory and preferentially targeted excitatory SDH neurons (^S1→^SDH neurons) broadly distributed across laminae I–V. ^S1→^SDH neurons in the superficial laminae also received excitatory inputs from both Aβ fibers and inhibitory inputs from neuropeptide Y promoter active SDH neurons (NpyP^+^ neurons). Furthermore, loss of inhibition from NpyP^+^ neurons induced Aβ fiber-derived allodynia, which was attenuated by suppressing descending signaling from S1^→SDH^ neurons to the SDH. Moreover, silencing ^S1→^SDH neurons alleviated neuropathic allodynia. These findings identify a new corticospinal mechanism that contributes to Aβ fiber-mediated neuropathic allodynia and highlight the S1→SDH pathway as a potential therapeutic target.

## 1. Introduction

Neuropathic pain frequently arises from injury to the somatosensory system associated with conditions such as cancer, chemotherapeutic treatment, surgery, or traumatic injury [1,2]. Mechanical allodynia is a principal symptom of neuropathic pain and is elicited by innocuous stimuli, such as light touch. Tactile information from the skin is conveyed to the spinal dorsal horn (SDH) by primary afferent low-threshold mechanoreceptors (LTMRs), including Aβ fibers [3,4,5]. The SDH is recognized as the first relay station in the central nervous system, serving as a crucial hub for processing somatosensory information and appropriately transmitting it to the brain [6,7,8]. Under physiological conditions, inputs transmitted by Aβ fibers do not activate pain-transmitting neurons located in lamina I of the SDH; however, under pathological conditions such as peripheral nerve injury (PNI), these Aβ fiber signals can lead to activation of these SDH neurons and potentially induce neuropathic pain [4,5,8,9,10]. Nevertheless, the mechanisms by which innocuous mechanical signals are converted into nociceptive outputs remain incompletely understood.

To elucidate these mechanisms, experimental tools that selectively activate primary afferent Aβ fibers in vivo are essential. We previously established an optogenetic approach to evaluate Aβ fiber-mediated neuropathic allodynia using the transgenic rat line W-TChR2V4 [11,12]. In these rats, channelrhodopsin-2 (ChR2) is expressed at the peripheral terminals of touch-sensing Aβ fibers in the skin [13], and blue light stimulation to the plantar skin of the rats subjected to PNI induces withdrawal behavior [11]. Furthermore, following PNI, optical stimulation of Aβ fibers activates SDH neurons in the superficial laminae, including lamina I, which are normally unresponsive to such input. More recently, we identified a population of inhibitory SDH interneurons captured by adeno-associated viral (AAV) vectors containing a neuropeptide Y promoter (referred to here as NpyP^+^ neurons) and demonstrated that loss of these neurons is sufficient to induce Aβ fiber-derived allodynia and that attenuation of their inhibitory signaling after PNI critically contributes to neuropathic allodynia [12]. Together, these findings suggest that maladaptive alterations in SDH circuitry after PNI are a central element in the pathological conversion of Aβ fiber-derived signals into pain.

In addition to the SDH, growing evidence indicates that neuropathic allodynia involves the anterior cingulate cortex (ACC) [14,15,16], the insular cortex (IC) [17], and the primary somatosensory (S1) cortex [18,19]. In S1 cortical neurons, PNI has been reported to induce dendritic spine remodeling as well as an increase in the number of c-FOS^+^ neurons [19,20]. A more recent study has shown that hyperactivity of vasoactive intestinal peptide-expressing neurons in the S1 cortex during non-rapid eye movement sleep is important for both the onset and maintenance of neuropathic pain [21]. Furthermore, anatomical studies, including recent comprehensive mapping analyses, have revealed that not only brainstem neurons but also neurons from higher cortical areas project their axons directly to the SDH [22,23,24,25,26,27,28,29]. Among them, the S1 cortex contains a substantial population of neurons that project to the SDH [26,27,28,29]. Moreover, SDH-projecting S1 cortical neurons (S1^→SDH^ neurons) have been implicated in PNI-induced mechanical allodynia in mice [18,30], suggesting that top-down signaling from S1^→SDH^ neurons may contribute to pathological reorganization of SDH circuits and the development of neuropathic allodynia. However, the functional connectivity between S1^→SDH^ neurons and SDH neural circuits, and their contribution to Aβ fiber-induced neuropathic allodynia, remains unknown.

To address this gap, we combined our established optogenetic model of allodynia (W-TChR2V4 rats) with circuit-specific electrophysiological recordings (whole-cell patch-clamp), cell ablation using the toxin receptor-mediated cell knockout (TRECK) system [31], and chemogenetic manipulation via multiple AAV vectors. Using these tools, we demonstrate that S1^→SDH^ neurons provide direct excitatory input to SDH neurons (^S1→^SDH neurons), which also receive excitatory and inhibitory inputs from primary afferent Aβ fibers and NpyP^+^ SDH neurons, respectively. Furthermore, we show that descending signaling from the S1 to the SDH is a critical driver of PNI-induced pathological neural activity within SDH circuits that underlies neuropathic allodynia, identifying the S1→SDH pathway as a potential therapeutic target.

## 2. Materials and Methods

### 2.1. Animals

W-Tg(Thy1-COP4/YFP∗) 4Jfhy (W-TChR2V4: NBRPRat No. 0685) rats [13,32] were supplied by the National BioResource Project—Rat, Kyoto University (Kyoto, Japan). All male W-TChR2V4 and Wistar rats (CLEA Japan, Tokyo, Japan) were aged 4–5 weeks at the start of each experiment and were housed in an individual cage at a temperature of 22 ± 1 °C with a 12-h light–dark cycle (light from 8:00 to 20:00) and fed food and water ad libitum. W-TChR2V4 rats were randomly allocated to each experimental group. The sample sizes were determined based on our prior studies employing similar methodologies [11,12]. Behavioral experiments were conducted without blinding the experimenter to group allocation. All animal experiments were conducted according to the national and international guidelines contained in the ‘Act on Welfare and Management of Animals’ (Ministry of Environment of Japan) and ‘Regulation of Laboratory Animals’ (Kyushu University) and under the protocols approved by the Institutional Animal Care and Use committee review panels at Kyushu University (the approval codes of this study are A24-468-0, 24 January 2025 and 6-111, 12 March 2025).

### 2.2. Recombinant Adeno-Associated Virus (rAAV) Vector Production

To produce rAAV vectors for NpyP-dependent gene transduction, we first generated a vector containing the NpyP (NCBI GenBank: HM443071.1; 1659 bp; –633 to +26 [+1 = transcription start site]) by replacing the CMV promoter in pZac2.1 (University of Pennsylvania Gene Therapy Program Vector Core) with the NpyP. We then cloned tdTomato, ChR2, diphtheria toxin receptor (DTR; kindly provided by Prof. Kenji Kohno, Nara Institute of Science and Technology), human muscarinic Gi-protein-coupled receptor fused to HA-tag (HA-hM4Di; #45548, Addgene, Watertown, MA, USA), human muscarinic Gq-protein-coupled receptor fused to HA-tag (HA-hM3Dq; #45547, Addgene), enhanced green fluorescent protein (EGFP; #RDB17866, Riken BRC, Saitama, Japan), green fluorescent protein derived from Aequorea coerulescens (acGFP; #632468, Takara Bio Inc., Shiga, Japan), enhanced synapsin promoter (ESYN), elongation factor 1α (EF1α), nlsCre, FLEx (#38042, Addgene), and mCherry into the modified pZac2.1 backbone to generate the following constructs: pZac2.1-EF1α-FLEx[acGFP]-WPRE, pZac2.1-ESYN-nlsCre-WPRE, pZac2.1-EF1α-FLEx[HA-hM4Di]-WPRE, pZac2.1-EF1α-FLEx[mCherry]-WPRE, pZac2.1-EF1α-FLEx[HA-hM3Dq]-WPRE, pZac2.1-NpyP-tdTomato-WPRE, pZac2.1-NpyP-ChR2-mCherry-WPRE, and pZac2.1-NpyP-DTR-EGFP-WPRE. rAAV vectors were produced from human embryonic kidney 293T (HEK293T) cells using a triple transfection protocol, as previously described [12]. Briefly, cis-plasmids (pZac or pAAV) were co-transfected with pAAV2/1 (#112862, Addgene), pAAV2/5, pAAV2/9 (University of Pennsylvania Gene Therapy Program Vector Core), or pAAV2/retro (#81070, Addgene), together with the adenoviral helper plasmid pAd DeltaF6 (University of Pennsylvania Gene Therapy Program Vector Core). Vectors were purified using two cesium chloride density gradient steps, followed by dialysis against phosphate-buffered saline (PBS; #041-20211; Wako, Saitama, Japan) containing 0.001% (*v*/*v*) Pluronic F-68 using Amicon Ultra-100 K filter units (Millipore, Darmstadt, Germany). The rAAV genome titer was determined using the PicoGreen fluorometric assay (Molecular Probes, Eugene, OR, USA) after denaturation of the viral particles. Purified vectors were aliquoted and stored at –80 °C until use.

### 2.3. Intra-SDH Injection of rAAV Vectors

Rats were anesthetized with 2% isoflurane and intraperitoneally (i.p.) injected with a mixture of medetomidine hydrochloride (0.15 mg/kg), midazolam (2 mg/kg), and butorphanol tartrate (2.5 mg/kg). As described in our previous studies [12], the skin over Th12–L3 was incised, and custom-made clamps were attached to the caudal side of the vertebral column. Paraspinal muscles around the left side of the interspace between Th13 and L1 vertebrae were removed. The dura mater and arachnoid membrane were carefully incised with the tip of a 30G needle to create a small window. A glass pipette backfilled with rAAV solution was inserted into the fourth lumbar (L4) segment of SDH (500 μm lateral from the midline and 250 μm in depth from the dorsal root entry zone), and 800 nL of rAAV solution was injected using a microsyringe pump (IMS-20, NARISHIGE, Tokyo, Japan) at 100 nL/min. After injection, the glass pipette was withdrawn, the skin was sutured with 3–0 silk, and gentamicin (0.5 mg/mL, i.p.) was administered to prevent infection. Rats were maintained under a heating light until recovery. Virus-injected rats were used for further analyses at least 3 weeks after the last injection. Viral titers were as follows: AAV2/retro-ESYN-nlsCre-WPRE, 2.0 × 10^12^ genome copies (GC)/mL; AAV2/5-EF1α-FLEx[acGFP]-WPRE, AAV2/9-EF1α-FLEx[mCherry]-WPRE, AAV2/9-NpyP-tdTomato-WPRE, AAV2/9-NpyP-ChR2-mCherry-WPRE, AAV2/9-NpyP-DTR-EGFP-WPRE, and AAV2/5-EF1α-FLEx[HA-hM4Di]-WPRE, 3.0 × 10^12^ GC/mL.

### 2.4. Intra-S1 Injection of rAAV Vectors

Under isoflurane (2%) anesthesia, rats were i.p. injected with three types of mixed anesthetic agents [medetomidine hydrochloride (0.15 mg/kg), midazolam (2 mg/kg), and butorphanol tartrate (2.5 mg/kg)]. According to the methods in our previous studies [12], after confirming the anesthetic effect, hair on the head was shaved and xylocaine (2%) was applied. After making an incision on the scalp, the head was fixed using a positioning fixture, and a portion of the skull (about 1 mm in diameter) on the contralateral where AAV microinjection was performed in the spinal cord was scraped with a dental drill to expose the dura mater. Then, the glass pipette backfilled with rAAV solution was inserted into the S1 (anteroposterior (AP): −2.0 mm from the bregma; mediolateral (ML): 2.0 mm (contralateral where AAV microinjection was performed in the SDH); dorsoventral (DV): −1.2 mm from the dura), and 1000 nl rAAV solution was microinjected using the microsyringe pump (100 μL/min). To keep the bregma and lambda in the same horizontal plane, tolerance was maintained at <50 µm in the dorsoventral axis between the bregma/lambda. After microinjection, the inserted glass pipette was removed from the S1, the skin was sutured with 3–0 silk, gentamicin (0.5 mg/mL) was administered i.p. to prevent infection, and the rats were kept under a heating light source until recovery. We used virus-injected rats for further analyses 3 weeks or more after the last injection. The following viral titers were used: AAV2/5-EF1α-FLEx[acGFP]-WPRE, AAV2/5-EF1α-FLEx[HA-hM4Di]-WPRE and AAV2/5-EF1α-FLEx[HA-hM3Dq]-WPRE: 3.0 × 10^12^ genome copies (GC)/mL, AAV2/1-ESYN-nlsCre-WPRE: 1.0 × 10^13^ genome copies (GC)/mL.

### 2.5. Immunohistochemistry

Rats were deeply anesthetized with an i.p. injection of pentobarbital and transcardially perfused with PBS followed by ice-cold 4% paraformaldehyde (PFA; #162-16065; Wako) in PBS. The transverse L4 segments of the spinal cord and brain were removed, postfixed in the same fixative for 3 h (spinal cord) or overnight (brain) at 4 °C, and cryoprotected in 30% sucrose for 24–48 h at 4 °C. Tissues were then embedded in OCT compound (#4583; Sakura Finetek Japan, Osaka, Japan) and stored at −80 °C. Transverse spinal cord and brain sections (30 μm) were incubated in blocking solution (3% normal goat serum [#S-1000; Vector Laboratories, Newark, CA, USA] or normal donkey serum [#017-000-121; Jackson ImmunoResearch, West Grove, PA, USA]) for 2 h at room temperature, followed by incubation with primary antibodies for 48 h at 4 °C: rabbit polyclonal anti-GFP (1:1000; #598; MBL International, Tokyo, Japan), anti-isolectin B4 (IB4)-biotin conjugate (1:1000; #I21414; Thermo Fisher, Waltham, MA, USA), rabbit polyclonal anti-GABA (1:1000; #A2052; Sigma-Aldrich, St. Louis, MO, USA), rabbit monoclonal anti-HA-tag (1:1000; #3724; Cell Signaling, Danvers, MA, USA), guinea pig monoclonal anti-c-FOS (1:10000; #226308; Synaptic Systems, Goettingen, Germany), goat polyclonal anti-paired box 2 (PAX2) (1:500; #AF3364; R&D Systems, Minneapolis, MN, USA), rat monoclonal anti-mCherry (1:2000; #M11217; Thermo Fisher), and goat polyclonal anti-DTR (1:500; #AF-259; R&D Systems). Sections were washed and incubated with appropriate secondary antibodies (Alexa Fluor 488, 546; #A21206, A11008, A11035, A11074, A11081, A31556, S32351; Thermo Fisher; #ab175678, Abcam, Cambridge, UK; DyLight 405, #711-165-152, Jackson ImmunoResearch) for 3 h at room temperature. After washing, sections were mounted on slides and coverslipped with VECTASHIELD with or without 4′,6-diamidino-2-phenylindole (DAPI; Vector Laboratories). Images were acquired using a confocal microscope (LSM700 and LSM900, Zeiss, Oberkochen, Germany). For c-FOS immunostaining in the SDH, W-TChR2V4 rats at 14 days post-PNI were habituated for 30 min, followed by i.p. administration of deschloroclozapine (DCZ) or vehicle. Sixty min later, blue light (5 Hz, 500 ms interval) was applied to the plantar surface for 10 min. Ninety min after optical stimulation, rats were deeply anesthetized with isoflurane, perfused as described above, and the L4 spinal cord was collected. Transverse sections (30 μm) were stained with anti-c-FOS (1:10,000) and IB4-biotin (1:1000). For quantification, three to four randomly selected sections per rat were analyzed. The number of c-FOS^+^ neurons in superficial laminae I–V was counted using ZEN lite (https://www.zeiss.com/microscopy/ja/products/software/zeiss-zen-lite.html (accessed on 24 November 2025); ZEISS) and ImageJ (https://imagej.net/ij/ (accessed on 24 November 2025)).

### 2.6. Neuropathic Pain Model and Behavioral Assays

A modified L5 spinal nerve injury model was used [33,34]. Under 2% isoflurane anesthesia, the left L5 spinal nerve was tightly ligated with 5–0 silk and transected distal to the ligature. Sham-operated rats underwent exposure of the transverse process of the lumbar vertebra without nerve ligation or transection. The wound and skin were sutured with 3–0 silk.

For assessing Aβ fiber-derived allodynia, W-TChR2V4 rats were placed on a transparent acrylic plate and habituated for 30–60 min [11,12]. The plantar surface of each hindpaw was illuminated through the acrylic plate with a blue laser diode (470 nm, 5 Hz, 10 s interval, 10 stimulations per hindpaw; COME2-LB473/532/100, Lucir, Osaka, Japan). Light power at the skin was 1 mW, measured using a thermopile (COME2-LPM-NOVA, Lucir). Paw withdrawal responses were scored as follows: 0, no reaction; 1, mild movement without lifting/flinching; 2, hindpaw lifting and flinching. Total scores were calculated from 10 trials per hindpaw. To ensure consistent responses, rats were awake, hindpaws placed on the plate, and the animals were at rest.

For von Frey test, mechanical sensitivity was assessed using calibrated von Frey filaments (1.0–15.0 g, Stoelting, IL, USA) applied to the plantar surface, and the 50% paw withdrawal threshold was determined [12,35].

### 2.7. Chemogenetic Manipulation of S1^→SDH^ Neurons

hM4Di or hM3Dq was expressed in S1^→SDH^ neurons using AAV vectors with Cre/FLEx systems [12,30]. Rats were subjected to PNI ≥ 21 days after AAV injection. Behavioral experiments were conducted ≥4 weeks post-AAV injection and 2 weeks after PNI. DCZ (0.5 mg/kg, i.p.) was administered to activate hM4Di or hM3Dq. In control groups, vehicle (1% DMSO) was administered. Light stimulation of the hindpaw and von Frey tests were performed and scored before and after DCZ or vehicle administration.

### 2.8. Whole-Cell Patch-Clamp Recordings

As previously described [12,36], under anesthesia with urethane (1.2–1.5 g/kg, i.p.), the lumbosacral spinal cord was removed from W-TChR2V4 and Wistar rats and placed into a cold, N-Methyl-D-glucamine (NMDG)-based artificial cerebrospinal fluid (aCSF) (93 mM NMDG, 2.5 mM KCl, 1.25 mM NaH_2_PO_4_, 30 mM NaHCO_3_, 20 mM HEPES, 25 mM glucose, 2 mM thiourea, 0.5 mM CaCl_2_, 10 mM MgSO_4_, 5 mM l-ascorbic acid, 3 mM sodium pyruvate, and 12 mM N-acetyl-l-cysteine). A parasagittal spinal cord slice (300–350 μm thick) with an attached L4 dorsal root was made using a vibrating microtome (VT1200, Leica, Tokyo, Japan), and the slices were first incubated in a holding chamber with cutting solution at 31–34 °C for 10 min. After this initial recovery period, the slices were maintained in oxygenated aCSF solution (125 mM NaCl, 2.5 mM KCl, 2 mM CaCl_2_, 1 mM MgCl_2_, 1.25 mM NaH_2_PO_4_, 26 mM NaHCO_3_, and 20 mM glucose) at room temperature (22–25 °C) for at least 30 min. The spinal cord slice was then put into a recording chamber where oxygenated aCSF solution (26–28 °C) was continuously superfused at a flow rate of 4–7 mL/min. SDH neurons were visualized with an upright microscope equipped with infrared differential interference contrast Nomarski (FN1, Nikon, Tokyo, Japan). The patch pipettes were filled with an internal solution (125 mM K-gluconate, 10 mM KCl, 0.5 mM EGTA, 10 mM HEPES, 4 mM ATP-Mg, 0.3 mM NaGTP, 10 mM phosphocreatine, pH 7.28 adjusted with KOH). The pipette tip resistance was 4–10 MΩ. Synaptic currents were recorded using a computer-controlled amplifier (Axopatch 700B, Molecular Devices, San Jose, CA, USA). The data were digitized with an analog-to-digital converter (Digidata 1550, Molecular Devices), stored on a personal computer using a data acquisition program (pCLAMP 11.1 acquisition software, Molecular Devices), and analyzed using a software package (Clampfit version 11.2, Molecular Devices). Excitatory postsynaptic currents (EPSCs) and inhibitory postsynaptic currents (IPSCs) were recorded in the voltage-clamp mode at a holding potential of −70 mV and −45 mV, respectively. Optical (blue light intensity, 4 V; duration, 5 ms) and electrical stimuli (intensity, <40 µA; duration, 0.1 ms) were applied to the dorsal root for Aβ fiber stimulation as described previously [12]. Light-evoked EPSCs were considered to be monosynaptic responses if they did not exhibit failures upon repetitive stimulation at 1 Hz and if they exhibited a low jitter (<1 ms) upon stimulation at 0.1 Hz [37,38]. The membrane potentials were recorded in the current-clamp mode, and the firing patterns were determined by passing 1.0 s depolarizing current pulses through the recording electrode at the resting membrane potential. Light-evoked IPSCs from NpyP^+^ neurons to ^S1→^SDH neurons were recorded with optical fiber placed over the spinal cord slice. Bicuculline (10 μM, Sigma-Aldrich) and strychnine (1 μM, Sigma-Aldrich) were bath-applied for 3 min before light stimulation of NpyP^+^ neurons.

### 2.9. Administration of Diphtheria Toxin (DTX)

Two weeks after intra-SDH injection of AAV2/9-NpyP-DTR-EGFP-WPRE into the L4 segment, diphtheria toxin (DTX; 50 μg/kg in PBS, #048-34371, Wako) was administered i.p. twice at 72-h intervals, as described previously [12]. Rats were used for subsequent analyses ≥6 weeks after the final DTX injection. At that time, our previous study confirmed that no glial activation was observed in the SDH [12].

### 2.10. Statistical Analysis

Statistical analyses were performed using the GraphPad Prism 7.01 software (GraphPad Software Inc., San Diego, CA, USA). Quantitative data were expressed as the mean ± SEM. Statistical analyses of the results were conducted with two-way repeated-measures ANOVA with post hoc Bonferroni multiple comparison tests, one-way ANOVA with post hoc Bonferroni multiple comparison tests and unpaired *t*-test. *p*-values are indicated as * *p* < 0.05, ** *p* < 0.01, *** *p* < 0.001, and **** *p* < 0.0001.

## 3. Results

### 3.1. S1^→SDH^ Neurons Contribute to Aβ Fiber-Derived Neuropathic Allodynia in Rats

**Figure 1:**
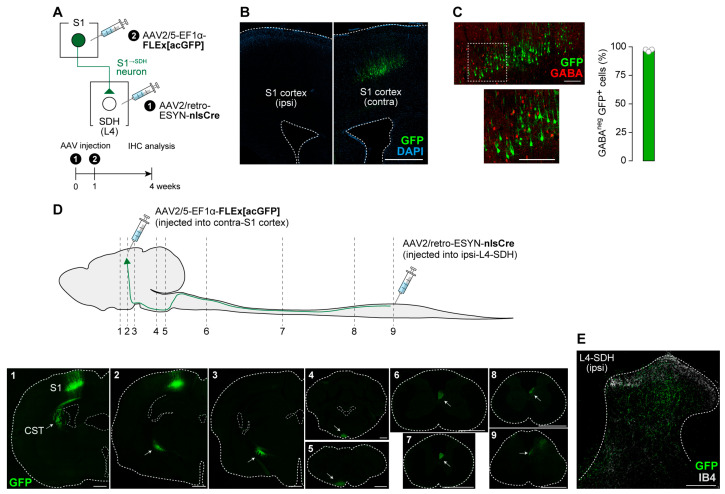
Intersectional gene expression in S1^→SDH^ neurons in rats. (**A**) Schematic diagram of the strategy used to selectively express genes in S1^→SDH^ neurons. AAV2/retro-ESYN-nlsCre was injected into the SDH, and 1–8 days later, AAV2/5-EF1α-FLEx[acGFP] was injected into the contralateral S1 of Wistar rats. (**B**) Representative images showing GFP expression in S1^→SDH^ neurons (green) in the ipsilateral and contralateral S1 cortex 4 weeks after AAV injection into the S1. Scale bar, 1000 μm. (**C**) Immunostaining of GABA in S1^→SDH^ neurons (GFP) in the contralateral S1 cortex. The percentage of GFP^+^ cells lacking GABA immunofluorescence was quantified (right) (*n* = 904 total GFP^+^ cells). Scale bars, 200 μm. (**D**) Schematic diagram of the axonal projection pathway of S1^→SDH^ neurons (GFP, green) to the lumbar SDH. Immunostaining images show somas and axons of S1^→SDH^ neurons in sections indicated by dotted lines. Arrows indicate the corticospinal trajectory (CST) pathway. Scale bar, 1000 μm. (**E**) Representative image of axonal fibers and terminals of S1^→SDH^ neurons (GFP, green) in the SDH of the L4 segment. Lamina IIi was visualized by IB4 staining (white). Scale bar, 200 μm.

To investigate the role of descending S1 cortical signaling to the SDH in neuropathic allodynia, we first established a strategy for S1^→SDH^ neuron-specific gene expression using AAV vectors in rats. By injecting AAV2/retro-ESYN-nlsCre into the SDH and AAV2/5-EF1α-FLEx[acGFP] into the contralateral S1 cortex (Figure 1A), GFP expression was restricted to the contralateral S1 cortex and was absent on the ipsilateral side (Figure 1B). Most GFP^+^ S1^→SDH^ neurons were not positive for GABA immunofluorescence (Figure 1C), indicating that these neurons are excitatory. GFP^+^ axons descended through the corticospinal tract (CST) to the spinal cord and terminated in the L4 segment of the SDH where AAV2/retro-ESYN-nlsCre was injected (Figure 1D,E). Within the SDH, axonal fibers and terminals were widely distributed in laminae I–IV (Figure 1E,F). These results confirmed that this AAV injection strategy enables selective gene expression in S1^→SDH^ neurons in rats.

**Figure 2:**
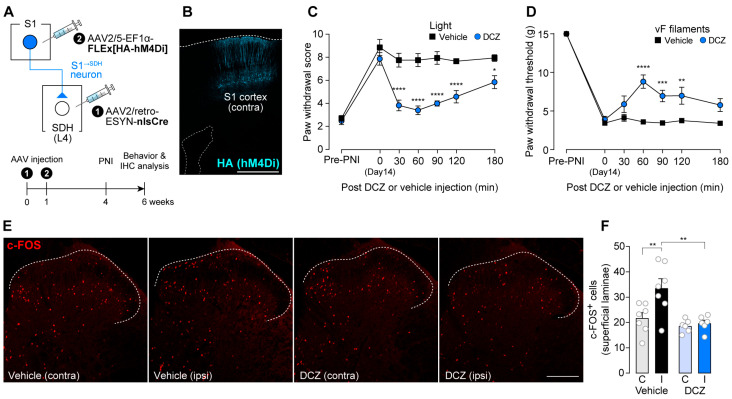
S1^→SDH^ neurons contribute to Aβ fiber-induced activation of SDH neurons and neuropathic allodynia in rats. (**A**) Schematic diagram of the strategy used to express hM4Di in S1^→SDH^ neurons. AAV2/retro-ESYN-nlsCre was injected into the SDH, and 1–8 days later, AAV2/5-EF1α-FLEx[HA-hM4Di] was injected into the contralateral S1 cortex of W-TChR2V4 rats. (**B**) Representative images showing expression of hM4Di (HA) in the S1 cortex 6 weeks after AAV injection into the S1. Scale bar, 1000 μm. (**C**,**D**) Paw withdrawal score to light stimulation (**C**) and withdrawal threshold to von Frey filaments (**D**) in W-TChR2V4 rats before (Pre-PNI) and 14 days after PNI. On day 14, vehicle or DCZ (0.5 mg/kg) was administered (vehicle, *n* = 11 rats; DCZ, *n* = 12 rats). * *p* < 0.05, ** *p* < 0.01, *** *p* < 0.001, and **** *p* < 0.0001 (repeated measures two-way ANOVA with post hoc Bonferroni multiple comparison test vs. vehicle group). (**E**,**F**) Representative images and quantification of c-FOS^+^ cells in the superficial laminae of the L4-SDH of W-TChR2V4 rats 14 days after PNI. Light stimulation to the hindpaw was applied at 30 min after vehicle or DCZ administration (vehicle, *n* = 7 rats; DCZ, *n* = 6 rats). Scale bar, 200 μm. ** *p* < 0.01 (one-way ANOVA with post hoc Bonferroni multiple comparison test). Data are presented as mean ± SEM.

To silence S1^→SDH^ neurons chemogenetically, we used hM4Di, inhibitory designer receptors exclusively activated by designer drugs (DREADD) [39]. AAV2/5-EF1α-FLEx[HA-hM4Di] was injected into the contralateral S1 cortex of W-TChR2V4 rats, which allow optogenetic activation of touch-sensing Aβ fibers, which had been injected with AAV2/retro-ESYN-nlsCre into the SDH (Figure 2A). As observed for GFP (Figure 1B), HA-hM4Di was expressed in the S1 cortex (Figure 2B). Consistent with previous studies [11,12,40], paw withdrawal responses to blue light stimulation of the plantar skin were markedly increased 14 days after PNI (Figure 2C), reflecting Aβ fiber-derived neuropathic allodynia-like behavior. A single administration of DCZ, an hM4Di agonist [41], at day 14-post PNI significantly suppressed this Aβ fiber-evoked behavioral response (Figure 2C). Similarly, behavioral hypersensitivity to mechanical stimulation of the plantar skin by von Frey filaments was also attenuated by DCZ treatment (Figure 2D). In addition, after PNI, blue light stimulation of Aβ fibers increased the number of c-FOS^+^ neurons in the superficial laminae of the SDH; this increase was significantly reduced by DCZ administration (Figure 2E,F). Together, these findings indicate that descending inputs from S1^→SDH^ neurons contribute to PNI-induced alterations in somatosensory processing within the SDH that underlie neuropathic allodynia.

### 3.2. S1^→SDH^ Neurons Form Synaptic Connections with SDH Neurons That Receive Aβ Fiber Input

**Figure 3:**
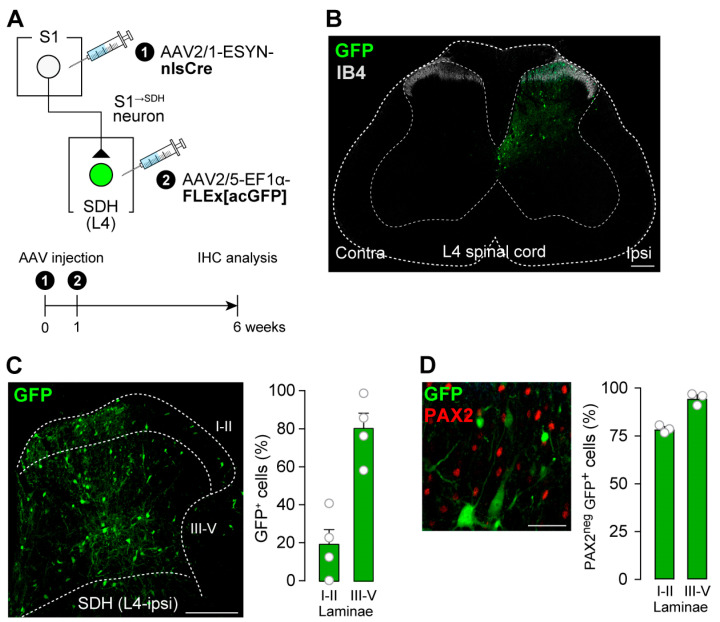
S1^→SDH^ neurons form synaptic connections with excitatory SDH neurons. (**A**) Schematic of ^S1→^SDH neuron labeling. AAV2/1-ESYN-nlsCre was injected into the S1 cortex, and 1–8 days later, AAV2/5-EF1α-FLEx[acGFP] was injected into the SDH of Wistar rats. (**B**) Representative images of ^S1→^SDH neurons (GFP) in the L4 spinal cord 4 weeks after AAV injection into the SDH. IB4 indicates lamina IIi. Scale bar, 200 μm. (**C**) Representative image and quantification of ^S1→^SDH neurons (GFP) in the ipsilateral L4-SDH. The distribution of GFP^+^ cells in laminae I–II and III–V was quantitatively analyzed (right) (*n* = 521 total GFP^+^ cells from four rats). Scale bar, 200 μm. (**D**) Representative images and quantification of PAX2 immunostaining in ^S1→^SDH neurons (GFP) in the L4-SDH (left). Percentages of PAX2^neg^ GFP^+^ cells in laminae I–II and III–V were quantitatively analyzed (right) (*n* = 428 total GFP^+^ cells from three rats). Scale bar, 50 μm. Data are presented as mean ± SEM.

To label SDH neurons that receive direct input from S1^→SDH^ neurons, AAV2/1-ESYN-nlsCre was injected into the contralateral S1 cortex, and AAV2/5-EF1α-FLEx[acGFP] was injected into the SDH (Figure 3A). GFP^+^ cells were observed in the SDH (Figure 3B), and quantification revealed that most were distributed across laminae III–V (Figure 3C). These GFP^+ S1→^SDH neurons were not immunolabeled with PAX2 (Figure 3D), a marker of inhibitory neurons [42,43], indicating that the majority are excitatory.

**Figure 4:**
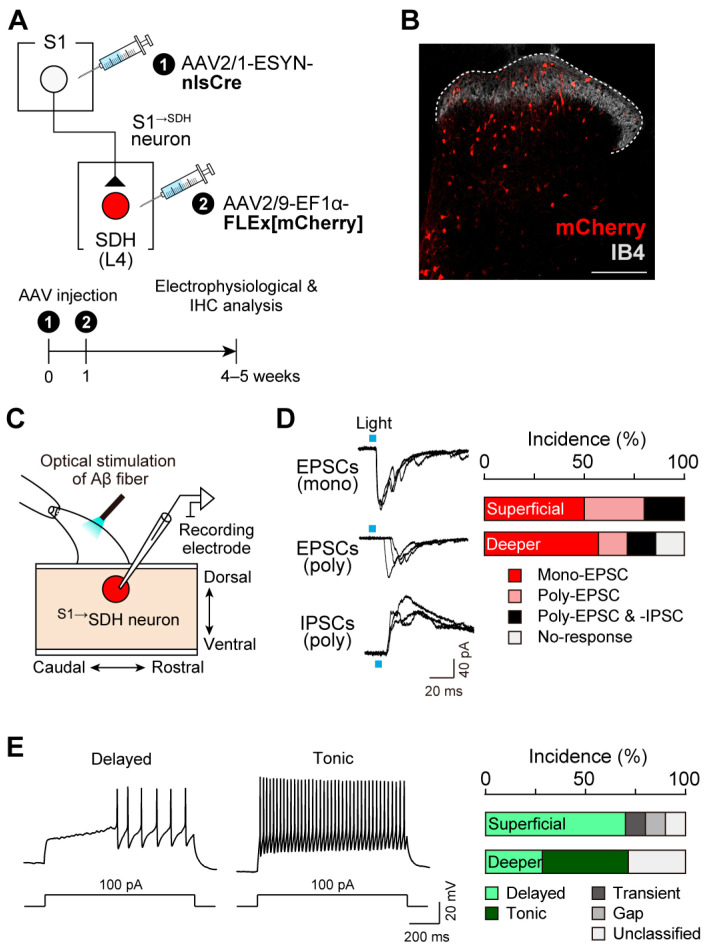
^S1→^SDH neurons receive excitatory inputs from primary afferent Aβ fibers. (**A**) Schematic of ^S1→^SDH neuron labeling. AAV2/1-ESYN-nlsCre was injected into the S1 cortex, and 1–8 days later, AAV2/9-EF1α-FLEx[mCherry] was injected into the SDH of W-TChR2V4 rats. (**B**) Representative images of ^S1→^SDH neurons (mCherry) in the ipsilateral L4-SDH 4 weeks after AAV injection into the SDH. IB4 indicates lamina IIi. Scale bar, 200 μm. (**C**) Schematic of whole-cell patch-clamp recordings from ^S1→^SDH neurons in parasagittal spinal cord slices with the L4 dorsal root from W-TChR2V4 rats. (**D**) Representative traces of mono- and polysynaptic EPSCs, and polysynaptic IPSCs in ^S1→^SDH neurons after light stimulation of Aβ fibers (0.1 Hz) (left), and the percentage of neurons displaying each synaptic input in superficial and deeper laminae (right) (*n* = 10 and 7 cells in superficial and deeper laminae, respectively). (**E**) Firing patterns of ^S1→^SDH neurons under current-clamp mode (left), and the percentage of neurons displaying each pattern in superficial and deeper laminae (right) (*n* = 10 and 7 cells in superficial and deeper laminae, respectively). Action potentials were evoked by current injection (100 pA).

To assess whether ^S1→^SDH neurons receive synaptic input from primary afferent Aβ fibers, we performed whole-cell patch-clamp recordings in spinal cord slices with intact dorsal roots from W-TChR2V4 rats. ^S1→^SDH neurons were visualized via mCherry expression by injecting AAV2/1-ESYN-nlsCre into the S1 cortex and AAV2/9-EF1α-FLEx[mCherry] into the SDH (Figure 4A,B). Stimulation of Aβ fibers by blue light illumination of the dorsal root evoked EPSCs and/or IPSCs in ^S1→^SDH neurons (Figure 4C,D). In superficial laminae, all ^S1→^SDH neurons tested responded to Aβ fiber stimulation; 50% of recorded neurons exhibited monosynaptic EPSCs (5/10 cells), and the remaining 50% displayed polysynaptic EPSCs (5/10 cells), with two of these also receiving polysynaptic IPSCs (Figure 4D). In deeper laminae, 57% of neurons received monosynaptic EPSCs and 29% received polysynaptic EPSCs, with one cell also exhibiting polysynaptic IPSCs (Figure 4D). The latencies (4.99 ± 0.44 and 11.41 ± 1.94 ms, *p* < 0.01) and jitters (0.354 ± 0.049 and 1.19 ± 0.256 ms, *p* < 0.01) of mono- and polysynaptic EPSCs, respectively, were statistically different. Furthermore, under the current-clamp mode, depolarizing current injection at the resting membrane potential elicited delayed firing patterns in most superficial ^S1→^SDH neurons (Figure 4E). Since delayed firing is characteristic of excitatory interneurons in superficial laminae [44], these observations support the excitatory identity of ^S1→^SDH neurons (Figure 3D). In contrast, ^S1→^SDH neurons in deeper laminae exhibited tonic, delayed, or unclassified firing patterns (Figure 4E). Collectively, these findings demonstrate that S1^→SDH^ neurons primarily form synaptic connections with excitatory SDH neurons in the superficial laminae that receive mono- and polysynaptic inputs from touch-sensing Aβ fibers.

### 3.3. Impact of S1^→SDH^ Neuron Signaling on Allodynia Is Unmasked Under Pathological Conditions

**Figure 5:**
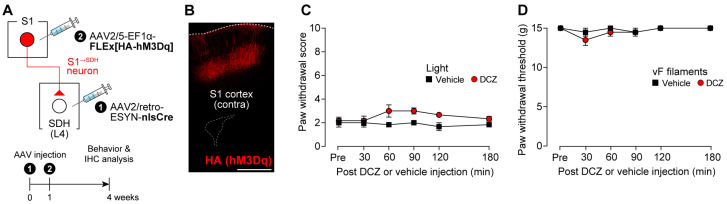
Activation of S1^→SDH^ neurons alone in normal rats does not induce Aβ fiber-mediated allodynia. (**A**) Schematic of the strategy to express hM3Dq in S1^→SDH^ neurons. AAV2/retro-ESYN-nlsCre was injected into the SDH, and 1–8 days later, AAV2/5-EF1α-FLEx[HA-hM3Dq] was injected into the contralateral S1 cortex of W-TChR2V4 rats. (**B**) Representative expression of hM3Dq (HA) in the contralateral S1 cortex 4 weeks after AAV injection into the S1. Scale bar, 1000 μm. (**C**,**D**) Paw withdrawal score by light (**C**) and threshold by von Frey filaments (**D**) of W-TChR2V4 rats before (Pre) and after administration of vehicle or DCZ (0.5 mg/kg) (*n* = 6 rats per group). Data were analyzed by repeated measures two-way ANOVA with post hoc Bonferroni’s multiple comparison test vs. the vehicle group. Data are presented as mean ± SEM.

Given that silencing S1^→SDH^ neurons suppressed neuropathic allodynia, we next examined whether activation of these neurons is sufficient to produce allodynia under normal conditions. To chemogenetically activate S1^→SDH^ neurons, AAV2/5-EF1α-FLEx[HA-hM3Dq] was injected into the contralateral S1 cortex of W-TChR2V4 rats that had received intra-SDH injections of AAV2/retro-ESYN-nlsCre (Figure 5A), and the excitatory DREADD hM3Dq (HA immunostaining) was expressed in S1^→SDH^ neurons (Figure 5B). However, DCZ administration did not alter the paw withdrawal response to light stimulation of Aβ fibers (Figure 5C), nor did it affect paw withdrawal thresholds in the von Frey test (Figure 5D). These findings indicate that activation of S1^→SDH^ neurons alone is insufficient to induce Aβ fiber-mediated allodynia under physiological conditions.

**Figure 6:**
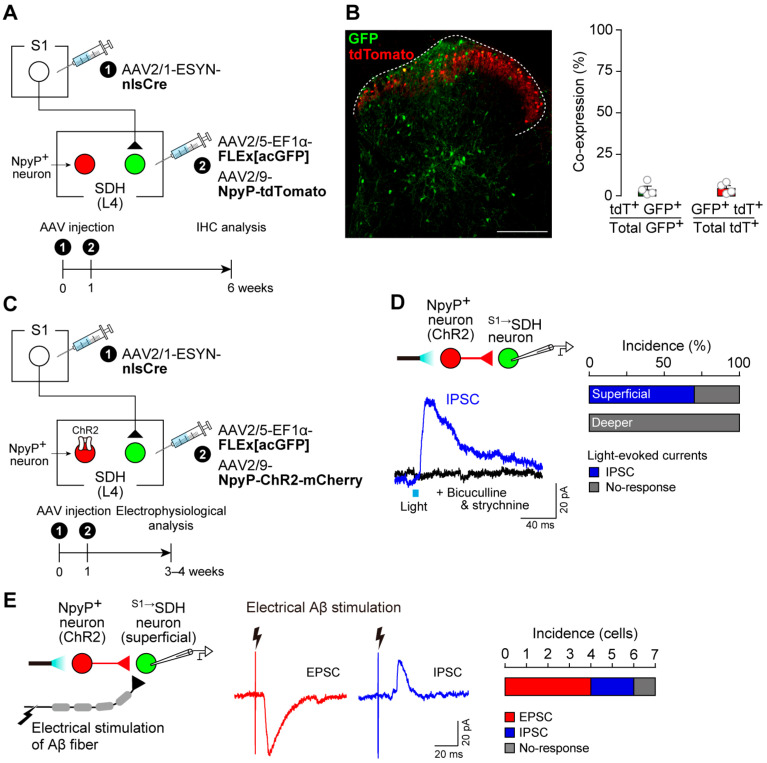
^S1→^SDH neurons receive inhibitory inputs from NpyP^+^ SDH neurons. (**A**) Schematic of labeling ^S1→^SDH neurons and NpyP^+^ neurons. AAV2/1-ESYN-nlsCre was injected into the S1 cortex, and 1–8 days later, AAV2/5-EF1α-FLEx[acGFP] and AAV2/9-NpyP-tdTomato were injected into the SDH of Wistar rats. (**B**) Representative images of ^S1→^SDH neurons (GFP) and NpyP^+^ neurons (tdTomato) in the L4-SDH 6 weeks after AAV injection into the SDH (left). Colocalization of tdTomato with GFP in laminae I–V was quantitatively analyzed (right) (*n* = 428 total tdTomato^+^ cells and *n* = 521 total GFP^+^ cells from four rats). Scale bar, 200 μm. (**C**) Schematic of GFP expression in ^S1→^SDH neurons and ChR2 expression in NpyP^+^ neurons. AAV2/1-ESYN-nlsCre was injected into the S1 cortex, and 1–8 days later, AAV2/5-EF1α-FLEx[acGFP] and AAV2/9-NpyP-ChR2-mCherry were injected into the SDH of Wistar rats. (**D**) Light-evoked IPSCs recorded from ^S1→^SDH neurons. Experimental schematic (left, top) and representative traces of light-evoked IPSCs (left, bottom) with or without bicuculline (10 µM) and strychnine (1 µM). The percentage of neurons displaying light-evoked IPSCs in superficial and deeper laminae is shown (*n* = 10 and 6 cells, respectively) (right). (**E**) Experimental schematic (left) and representative traces of electrically evoked Aβ fiber-mediated EPSCs and IPSCs in superficial ^S1→^SDH neurons receiving IPSCs from NpyP^+^ neurons (middle). The percentage of neurons displaying Aβ fiber-evoked EPSCs and IPSCs is shown (right) (*n* = 7 cells). Data are presented as mean ± SEM.

These observations suggested that the functional contribution of descending S1^→SDH^ signaling to neuropathic allodynia emerges under pathological conditions. We previously demonstrated that dysfunction of inhibitory NpyP^+^ neurons is directly involved in the pathological conversion of Aβ fiber signals into pain-like behavior [12]. Thus, we hypothesized that interactions between NpyP^+^ neurons and S1^→SDH^ signaling underlie this phenomenon. First, we examined whether S1^→SDH^ neurons directly innervate NpyP^+^ neurons. Rats were injected such that ^S1→^SDH neurons and NpyP^+^ neurons expressed GFP and tdTomato, respectively (Figure 6A). We observed virtually no overlap between these neuronal populations in the SDH (Figure 6B), suggesting that direct projections from S1 cortical neurons to NpyP^+^ neurons are minimal. Next, to assess the reverse connectivity, we expressed ChR2 in NpyP^+^ neurons (Figure 6C) and recorded IPSCs from ^S1→^SDH neurons in spinal cord slices following optogenetic stimulation (Figure 6D). Light activation of NpyP^+^ neurons evoked IPSCs in 70% of superficial ^S1→^SDH neurons (7/10 cells) (Figure 6D, right). These IPSCs were abolished by the GABA_A_ and glycine receptor antagonists bicuculline and strychnine, respectively (Figure 6D, left). In contrast, ^S1→^SDH neurons located in deeper laminae did not receive synaptic input from NpyP^+^ neurons (Figure 6D, right). Furthermore, superficial ^S1→^SDH neurons receiving inhibitory input from NpyP^+^ neurons also exhibited EPSCs and, to a lesser extent, IPSCs elicited by Aβ fiber stimulation via electrical dorsal root activation (25 μA) (Figure 6E). These electrophysiological results indicate that superficial ^S1→^SDH neurons integrate both excitatory inputs from Aβ fibers and inhibitory inputs from NpyP^+^ SDH neurons.

**Figure 7:**
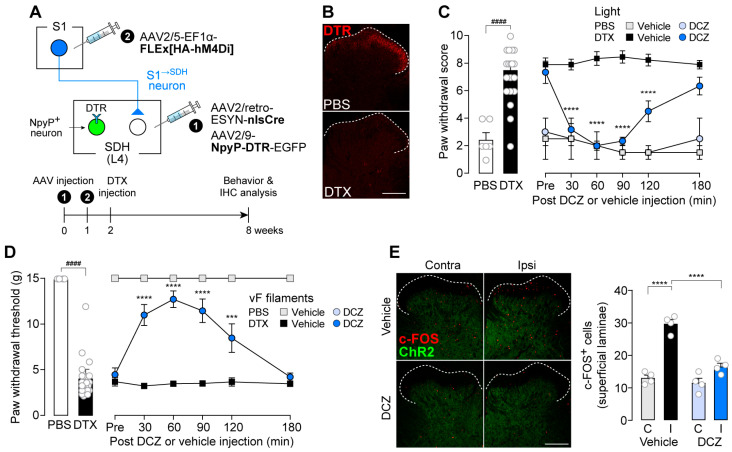
Silencing of S1^→SDH^ neurons suppresses Aβ fiber-induced activation in superficial SDH neurons and allodynia in rats with loss of NpyP^+^ SDH neurons. (**A**) Schematic of the strategy to specifically express hM4Di in S1^→SDH^ neurons and DTR in NpyP^+^ neurons. AAV2/retro-ESYN-nlsCre and AAV2/9-NpyP-DTR-EGFP were injected into the SDH, and 1–8 days later, AAV2/5-EF1α-FLEx[HA-hM4Di] was injected into the contralateral S1 cortex of W-TChR2V4 rats. (**B**) Representative DTR immunofluorescence in the L4-SDH after administration of DTX (50 µg/kg, i.p., two injections 72-h apart) or vehicle (PBS). Scale bar, 200 μm. (**C**,**D**) Paw withdrawal score by light (**C**) and threshold by von Frey filaments (**D**) of W-TChR2V4 rats. Six weeks after PBS or DTX administration (PBS, *n* = 6 rats; DTX, *n* = 21 rats), the effects of vehicle or DCZ (0.5 mg/kg) on behavioral responses were tested (PBS-DCZ, *n* = 3 rats; PBS-Vehicle, *n* = 3 rats; DTX-DCZ, *n* = 12 rats; DTX-Vehicle, *n* = 9 rats). #### *p* < 0.0001 (unpaired t-test); *** *p* < 0.001, and **** *p* < 0.0001 (repeated measures two-way ANOVA with post hoc Bonferroni’s multiple comparison test versus the DTX-Vehicle group). (**E**) Representative images and quantification of c-FOS^+^ cells in the L4-SDH (superficial laminae) of W-TChR2V4 rats 6 weeks after light stimulation to the hindpaw was applied at 30 min after vehicle or DCZ administration (*n* = 4 rats per group). Scale bar, 200 μm. **** *p* < 0.0001 (one-way ANOVA with post hoc Bonferroni multiple comparison test). Data are presented as mean ± SEM.

We therefore predicted that disruption of the excitatory/inhibitory (E/I) balance in ^S1→^SDH neurons contributes to their involvement in neuropathic allodynia. To test this hypothesis, we used rats in which NpyP^+^ neurons were ablated via the TRECK system (Figure 7A). Consistent with our previous findings [12], DTR expression in NpyP^+^ neurons was confirmed in rats receiving intra-SDH injections of AAV2/9-NpyP-DTR-EGFP (Figure 7B, upper), and ablation was achieved by two injections of DTX (50 μg/kg) administered 72-h apart (Figure 7B, lower). Loss of NpyP^+^ neurons increased paw withdrawal behavior in response to optogenetic activation of Aβ fibers (Figure 7C, left). This Aβ fiber-induced allodynia in NpyP^+^ neuron-ablated rats was suppressed by DCZ-induced chemogenetic silencing of S1^→SDH^ neurons (Figure 7C, right). Similar effects were observed in von Frey testing (Figure 7D). Moreover, optogenetic Aβ fiber stimulation increased the number of c-FOS^+^ neurons in the superficial SDH of NpyP^+^ neuron-ablated rats, and this increase was attenuated by DCZ-induced silencing of S1^→SDH^ neurons (Figure 7E). These results suggest that the functional influence of descending S1^→SDH^ signaling becomes unmasked when inhibitory input from NpyP^+^ neurons is lost, a phenomenon that occurs after PNI [12].

**Figure 8:**
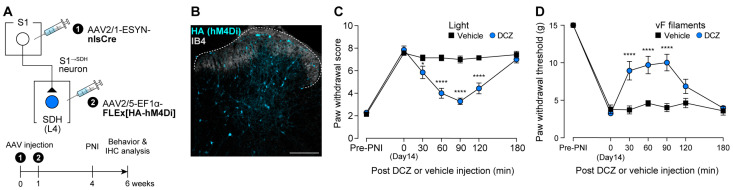
Silencing of ^S1→^SDH neurons alleviates Aβ fiber-induced neuropathic allodynia. (**A**) Schematic of the strategy to specifically express hM4Di in ^S1→^SDH neurons. AAV2/1-ESYN-nlsCre was injected into the S1 cortex, and 1–8 days later, AAV2/5-EF1α-FLEx[HA-hM4Di] was injected into the SDH of W-TChR2V4 rats. (**B**) Representative expression of hM4Di in ^S1→^SDH neurons (HA) in the ipsilateral L4-SDH 6 weeks after AAV injection into the SDH. IB4 indicates lamina IIi. Scale bar, 200 μm. (**C**,**D**) Paw withdrawal score by light (C) and threshold by von Frey filaments (D) of W-TChR2V4 rats before (Pre-PNI) and 14 days after PNI, and after administration of vehicle or DCZ (0.5 mg/kg) at day 14 post-PNI (*n* = 7 rats per group). * *p* < 0.05, **** *p* < 0.0001 (repeated measures two-way ANOVA with post hoc Bonferroni’s multiple comparison test vs. the vehicle group). Data are presented as mean ± SEM.

Finally, we further assessed the role of ^S1→^SDH neurons by inhibiting them chemogenetically during PNI-induced allodynia. AAV2/1-ESYN-nlsCre was injected into the contralateral S1 cortex, and AAV2/5-EF1α-FLEx[HA-hM4Di] was injected into the SDH (Figure 8A). HA (hM4Di) expression in ^S1→^SDH neurons was confirmed (Figure 8B). Chemogenetic silencing of ^S1→^SDH neurons of PNI rats using DCZ significantly reduced neuropathic allodynia evoked by optical Aβ fiber stimulation (Figure 8C) and similarly alleviated hypersensitivity to mechanical stimulation by von Frey filaments (Figure 8D). Collectively, these findings demonstrate that SDH neurons directly receiving descending input from the S1 cortex play a critical role in PNI-induced maladaptive processing in the SDH and neuropathic allodynia.

## 4. Discussion

Maladaptive alterations in somatosensory information processing within the SDH after PNI have long been investigated to elucidate the mechanisms underlying neuropathic pain. In particular, excitation of nociceptive transmission neurons in the SDH by primary afferent Aβ fibers has been considered a potential mechanism for neuropathic allodynia [1,4,6,8,9,10]. It is also well known that somatosensory information processing in the SDH is regulated by descending neural signals from the brain [1,7,45]. In this study, by combining AAV-based intersectional gene expression for the visualization and functional manipulation of S1 and SDH neurons with our established optogenetic model of allodynia (W-TChR2V4 rats), we demonstrate for the first time that descending signaling from S1 cortical neurons plays a crucial role in aberrant somatosensory processing within the SDH after PNI and in neuropathic allodynia. These findings provide new insights into corticospinal mechanisms underlying neuropathic pain.

Anatomical studies, including recent whole-brain imaging analyses, have identified several brain regions whose neurons project directly to the spinal cord [22,23,24,25,26,27,28,29]. Among these, the S1 cortex contains the largest number of such descending neurons, presuming their substantial impact on sensory processing in the SDH. Our study showed that chemogenetic silencing of predominantly excitatory S1^→SDH^ neurons suppressed the Aβ fiber-induced increase in c-FOS^+^ SDH neurons after PNI and alleviated neuropathic allodynia in rats. The involvement of S1^→SDH^ neurons in neuropathic allodynia is supported by previous studies using surgical transection or chemogenetic inhibition of the corticospinal tract in mice [18,30]. Furthermore, using an AAV2/1 vector, we visualized SDH neurons that receive direct synaptic inputs from S1 neurons and found that these ^S1→^SDH neurons are excitatory and broadly distributed across laminae I–V. This distribution is consistent with the localization of S1^→SDH^ axonal fibers and terminals predominantly within these laminae. We further demonstrated that ^S1→^SDH neurons receive excitatory inputs from Aβ fibers, supporting the link between S1→SDH signaling and allodynia. However, under physiological conditions, chemogenetic activation of descending S1 cortical neurons did not alter Aβ fiber-evoked behavior. This result suggests that excitatory signals from the S1 cortex remain functionally silent under normal conditions but may become unmasked in pathological states, exerting a substantial impact on SDH processing that leads to Aβ fiber-induced allodynia. Supporting this notion, inhibition of descending S1^→SDH^ neuronal signaling effectively suppressed Aβ fiber-evoked allodynia caused by the loss of inhibition from NpyP^+^ SDH neurons. NpyP^+^ neurons have been identified as gate-control cells that prevent tactile Aβ fiber signals from being transmitted to pain-transmitting neurons, and their functional impairment has been linked to neuropathic allodynia [12]. Given that ^S1→^SDH neurons receive inhibitory synaptic input from NpyP^+^ neurons, ^S1→^SDH neurons appear to be downstream targets of inhibitory NpyP^+^ neurons, and disruption of this inhibition may shift the E/I balance toward excitation. Under such conditions—especially those arising after PNI—the excitatory influence of descending S1 neuron signals on SDH sensory processing may become unmasked, leading to Aβ fiber-evoked allodynia. This view is further supported by the observation that inhibition of descending S1 neurons reduces the number of c-FOS^+^ neurons in the superficial SDH.

Chemogenetic silencing of S1^→SDH^ neurons suppressed neuropathic allodynia; however, it should be noted that this manipulation also inhibited ^S1→^SDH neurons located in deeper SDH regions, which do not receive synaptic input from NpyP^+^ neurons. Nonetheless, our previous report showed that functional suppression or activation of NpyP^+^ neurons did not alter the number of c-FOS^+^ neurons in the deep SDH [12]. Thus, it is conceivable that ^S1→^SDH neurons located in the superficial SDH may represent critical targets of descending S1 signaling that contribute to neuropathic allodynia, although the functions of ^S1→^SDH neurons in deeper laminae remain to be clarified.

Based on these findings, we propose the following neural circuit model. We hypothesize that under pathological conditions such as PNI, in which NpyP^+^ neuron activity is reduced [12], excitatory descending signals from S1^→SDH^ neurons may increase the responsiveness of ^S1→^SDH neurons to Aβ fiber inputs. Such a change could potentially contribute to the activation of pain-related neurons in the superficial SDH including lamina I neurons, although direct in vivo evidence for this mechanism remains to be established. Furthermore, recent studies have shown that PNI induces dendritic spine remodeling and increases the number of c-FOS^+^ neurons in the S1 cortex, and that suppression of these changes attenuates neuropathic mechanical hypersensitivity [19,20,46,47]. While our study did not directly assess S1 activity in vivo, these previous findings raise the possibility that descending signals from S1 neurons to the SDH may be altered after PNI in a manner that could increase the excitability of ^S1→^SDH neurons. Although characterization of gene expression and subpopulations of ^S1→^SDH neurons is an important future direction, these neurons may correspond to the cholecystokinin-expressing SDH neurons shown to receive inputs from both Aβ fibers and descending S1 neurons [18]. To define the gene expression profiles of these neurons, transcriptomic approaches would provide the most informative strategy. Moreover, previous studies have shown that enhanced glutamatergic activity from the ventral posterior lateral thalamic nucleus to the S1 cortex contributes to mechanical allodynia after PNI [48], and that increased projections from the posterior IC to the basolateral amygdala, as well as from the posterior IC to the ventromedial thalamic nucleus, are associated with neuropathic hyperalgesia [17]. These findings suggest that a broader network of S1 cortical neurons, involving interactions with other brain regions in addition to the SDH, may play a role in neuropathic allodynia.

There are several limitations to our study. First, consistent with our previous studies [11,12], only male rats were used as an initial step in examining the role of S1→SDH signaling in neuropathic allodynia. In addition, the use of a single neuropathic pain model and the focus primarily on a 14-day post-injury time point also limit the generalizability of our findings. Thus, an important direction for future research is to elucidate potential sex- and species-dependent differences and to examine the role of S1→SDH signaling in additional neuropathic pain models across a broader temporal window. Furthermore, our study was specifically designed to investigate neuropathic allodynia, particularly that induced by Aβ fiber activation. Therefore, assessing other pain-related behaviors—including spontaneous or ongoing pain and thermal hypersensitivity—will also be an important focus for future studies.

## 5. Conclusions

Our study, which revealed a new mechanism in the SDH whereby corticospinal signaling induces Aβ fiber-mediated neuropathic allodynia, leads to the possibility that pathological functional alterations in both the S1 cortex and SDH establish an aberrant brain–SDH network loop that plays a pivotal role in neuropathic allodynia. Thus, our study highlights the S1→SDH signaling pathway as a potential therapeutic target.

## Data Availability

The original contributions presented in this study are included in the article. Further inquiries can be directed to the corresponding author.

## Abbreviations

PNI	Peripheral nerve injury
SDH	Spinal dorsal horn
S1	Primary somatosensory
S1^→SDH^ neurons	Descending neurons projecting directly from the S1 cortex to the SDH
^S1→^SDH neurons	SDH neurons directly receiving projections from S1
NpyP	Neuropeptide Y promoter
LTMRs	Low-threshold mechanoreceptors
ChR2	Channelrhodopsin-2
AAV	Adeno-associated virus
ACC	Anterior cingulate cortex
IC	Insular cortex
TRECK	Toxin receptor-mediated cell knockout
rAAV	Recombinant adeno-associated virus
DTR	Diphtheria toxin receptor
HA	Hemagglutinin
hM4Di	Human muscarinic Gi-coupled receptor
hM3Dq	Human muscarinic Gq-coupled receptor
GFP	Green fluorescent protein
acGFP	Aequorea coerulescens GFP
ESYN	Enhanced synapsin
EF1α	Elongation factor 1α
WPRE	Woodchuck hepatitis virus post-transcriptional response element
HEK293T	Human embryonic kidney 293T
PBS	Phosphate-buffered saline
i.p.	Intraperitoneal
Th12	The twelfth thoracic
L4	The fourth lumbar
GC	Genome copy
AP	Anteroposterior
ML	Mediolateral
DV	Dorsoventral
PFA	Paraformaldehyde
IB4	Isolectin B4
GABA	γ-aminobutyric acid
PAX2	Paired box 2
DAPI	4′,6-diamidino-2-phenylindole
DCZ	Deschloroclozapine
DMSO	Dimethyl sulfoxide
NMDG	N-Methyl-D-glucamine
aCSF	Artificial cerebrospinal fluid
EPSCs	Excitatory postsynaptic currents
IPSCs	Inhibitory postsynaptic currents
DTX	Diphtheria toxin
SEM	Standard error of the mean
CST	Corticospinal tract
DREADD	Designer receptors exclusively activated by designer drugs
E/I	Excitatory/Inhibitory

## References

[1] Colloca L., Ludman T., Bouhassira D., Baron R., Dickenson A.H., Yarnitsky D., Freeman R., Truini A., Attal N., Finnerup N.B. (2017). Neuropathic pain. Nat. Rev. Dis. Primers.

[2] Soliman N., Moisset X., Ferraro M.C., de Andrade D.C., Baron R., Belton J., Bennett D.L.H., Calvo M., Dougherty P., Gilron I. (2025). Pharmacotherapy and non-invasive neuromodulation for neuropathic pain: A systematic review and meta-analysis. Lancet Neurol..

[3] Todd A.J. (2010). Neuronal circuitry for pain processing in the dorsal horn. Nat. Rev. Neurosci..

[4] Peirs C., Seal R.P. (2016). Neural circuits for pain: Recent advances and current views. Science.

[5] Tsuda M. (2019). New approach for investigating neuropathic allodynia by optogenetics. Pain.

[6] Koch S.C., Acton D., Goulding M. (2018). Spinal circuits for touch, pain, and itch. Annu. Rev. Physiol..

[7] Moehring F., Halder P., Seal R.P., Stucky C.L. (2018). Uncovering the cells and circuits of touch in normal and pathological settings. Neuron.

[8] Peirs C., Dallel R., Todd A.J. (2020). Recent advances in our understanding of the organization of dorsal horn neuron populations and their contribution to cutaneous mechanical allodynia. J. Neural Transm..

[9] Kuner R., Flor H. (2016). Structural plasticity and reorganisation in chronic pain. Nat. Rev. Neurosci..

[10] Hughes D.I., Todd A.J. (2020). Central nervous system targets: Inhibitory interneurons in the spinal cord. Neurotherapeutics.

[11] Tashima R., Koga K., Sekine M., Kanehisa K., Kohro Y., Tominaga K., Matsushita K., Tozaki-Saitoh H., Fukazawa Y., Inoue K. (2018). Optogenetic activation of non-nociceptive aβ fibers induces neuropathic pain-like sensory and emotional behaviors after nerve injury in rats. eNeuro.

[12] Tashima R., Koga K., Yoshikawa Y., Sekine M., Watanabe M., Tozaki-Saitoh H., Furue H., Yasaka T., Tsuda M. (2021). A subset of spinal dorsal horn interneurons crucial for gating touch-evoked pain-like behavior. Proc. Natl. Acad. Sci. USA.

[13] Ji Z.G., Ito S., Honjoh T., Ohta H., Ishizuka T., Fukazawa Y., Yawo H. (2012). Light-evoked somatosensory perception of transgenic rats that express channelrhodopsin-2 in dorsal root ganglion cells. PLoS ONE.

[14] Koga K., Descalzi G., Chen T., Ko H.G., Lu J., Li S., Son J., Kim T., Kwak C., Huganir R.L. (2015). Coexistence of two forms of ltp in acc provides a synaptic mechanism for the interactions between anxiety and chronic pain. Neuron.

[15] Chen T., Taniguchi W., Chen Q.Y., Tozaki-Saitoh H., Song Q., Liu R.H., Koga K., Matsuda T., Kaito-Sugimura Y., Wang J. (2018). Top-down descending facilitation of spinal sensory excitatory transmission from the anterior cingulate cortex. Nat. Commun..

[16] Song Q., Wei A., Xu H., Gu Y., Jiang Y., Dong N., Zheng C., Wang Q., Gao M., Sun S. (2024). An acc-vta-acc positive-feedback loop mediates the persistence of neuropathic pain and emotional consequences. Nat. Neurosci..

[17] Chen J., Gao Y., Bao S.T., Wang Y.D., Jia T., Yin C., Xiao C., Zhou C. (2024). Insula→amygdala and insula→thalamus pathways are involved in comorbid chronic pain and depression-like behavior in mice. J. Neurosci..

[18] Liu Y., Latremoliere A., Li X., Zhang Z., Chen M., Wang X., Fang C., Zhu J., Alexandre C., Gao Z. (2018). Touch and tactile neuropathic pain sensitivity are set by corticospinal projections. Nature.

[19] Danjo Y., Shigetomi E., Hirayama Y.J., Kobayashi K., Ishikawa T., Fukazawa Y., Shibata K., Takanashi K., Parajuli B., Shinozaki Y. (2022). Transient astrocytic mglur5 expression drives synaptic plasticity and subsequent chronic pain in mice. J. Exp. Med..

[20] Kim S.K., Hayashi H., Ishikawa T., Shibata K., Shigetomi E., Shinozaki Y., Inada H., Roh S.E., Kim S.J., Lee G. (2016). Cortical astrocytes rewire somatosensory cortical circuits for peripheral neuropathic pain. J. Clin. Investig..

[21] Zhou H., Li M., Zhao R., Sun L., Yang G. (2023). A sleep-active basalocortical pathway crucial for generation and maintenance of chronic pain. Nat. Neurosci..

[22] Basbaum A.I., Clanton C.H., Fields H.L. (1976). Opiate and stimulus-produced analgesia: Functional anatomy of a medullospinal pathway. Proc. Natl. Acad. Sci. USA.

[23] Basbaum A.I., Fields H.L. (1979). The origin of descending pathways in the dorsolateral funiculus of the spinal cord of the cat and rat: Further studies on the anatomy of pain modulation. J. Comp. Neurol..

[24] Leong S.K., Shieh J.Y., Wong W.C. (1984). Localizing spinal-cord-projecting neurons in adult albino rats. J. Comp. Neurol..

[25] Nudo R.J., Masterton R.B. (1988). Descending pathways to the spinal cord: A comparative study of 22 mammals. J. Comp. Neurol..

[26] Liang H., Paxinos G., Watson C. (2011). Projections from the brain to the spinal cord in the mouse. Brain Struct. Funct..

[27] Wang Z., Maunze B., Wang Y., Tsoulfas P., Blackmore M.G. (2018). Global connectivity and function of descending spinal input revealed by 3d microscopy and retrograde transduction. J. Neurosci..

[28] Winter C.C., Jacobi A., Su J., Chung L., van Velthoven C.T.J., Yao Z., Lee C., Zhang Z., Yu S., Gao K. (2023). A transcriptomic taxonomy of mouse brain-wide spinal projecting neurons. Nature.

[29] Xie J., Feng R., Chen Y., Gao L. (2023). Morphological analysis of descending tracts in mouse spinal cord using tissue clearing, tissue expansion and tiling light sheet microscopy techniques. Sci. Rep..

[30] Fujimori K., Sekine M., Watanabe M., Tashima R., Tozaki-Saitoh H., Tsuda M. (2022). Chemogenetic silencing of spinal cord-projecting cortical neurons attenuates aβ fiber-derived neuropathic allodynia in mice. Neurosci. Res..

[31] Saito M., Iwawaki T., Taya C., Yonekawa H., Noda M., Inui Y., Mekada E., Kimata Y., Tsuru A., Kohno K. (2001). Diphtheria toxin receptor-mediated conditional and targeted cell ablation in transgenic mice. Nat. Biotechnol..

[32] Tomita H., Sugano E., Fukazawa Y., Isago H., Sugiyama Y., Hiroi T., Ishizuka T., Mushiake H., Kato M., Hirabayashi M. (2009). Visual properties of transgenic rats harboring the channelrhodopsin-2 gene regulated by the thy-1.2 promoter. PLoS ONE.

[33] Ho Kim S., Mo Chung J. (1992). An experimental model for peripheral neuropathy produced by segmental spinal nerve ligation in the rat. Pain.

[34] Tsuda M., Shigemoto-Mogami Y., Koizumi S., Mizokoshi A., Kohsaka S., Salter M.W., Inoue K. (2003). P2x4 receptors induced in spinal microglia gate tactile allodynia after nerve injury. Nature.

[35] Tsuda M., Kohro Y., Yano T., Tsujikawa T., Kitano J., Tozaki-Saitoh H., Koyanagi S., Ohdo S., Ji R.R., Salter M.W. (2011). Jak-stat3 pathway regulates spinal astrocyte proliferation and neuropathic pain maintenance in rats. Brain.

[36] Sueto D., I E., Onishi A., Tsuda M. (2025). Spinal dorsal horn neurons involved in the alleviating effects of cannabinoid receptor agonists on neuropathic allodynia-like behaviors in rats. J. Pharmacol. Sci..

[37] Petreanu L., Huber D., Sobczyk A., Svoboda K. (2007). Channelrhodopsin-2-assisted circuit mapping of long-range callosal projections. Nat. Neurosci..

[38] Honsek S.D., Seal R.P., Sandkühler J. (2015). Presynaptic inhibition of optogenetically identified vglut3+ sensory fibres by opioids and baclofen. Pain.

[39] Roth B.L. (2016). Dreadds for neuroscientists. Neuron.

[40] Ishibashi T., Yoshikawa Y., Sueto D., Tashima R., Tozaki-Saitoh H., Koga K., Yamaura K., Tsuda M. (2022). Selective involvement of a subset of spinal dorsal horn neurons operated by a prodynorphin promoter in aβ fiber-mediated neuropathic allodynia-like behavioral responses in rats. Front. Mol. Neurosci..

[41] Nagai Y., Miyakawa N., Takuwa H., Hori Y., Oyama K., Ji B., Takahashi M., Huang X.P., Slocum S.T., DiBerto J.F. (2020). Deschloroclozapine, a potent and selective chemogenetic actuator enables rapid neuronal and behavioral modulations in mice and monkeys. Nat. Neurosci..

[42] Cheng L., Arata A., Mizuguchi R., Qian Y., Karunaratne A., Gray P.A., Arata S., Shirasawa S., Bouchard M., Luo P. (2004). Tlx3 and tlx1 are post-mitotic selector genes determining glutamatergic over gabaergic cell fates. Nat. Neurosci..

[43] Koga K., Kanehisa K., Kohro Y., Shiratori-Hayashi M., Tozaki-Saitoh H., Inoue K., Furue H., Tsuda M. (2017). Chemogenetic silencing of gabaergic dorsal horn interneurons induces morphine-resistant spontaneous nocifensive behaviours. Sci. Rep..

[44] Yasaka T., Tiong S.Y.X., Hughes D.I., Riddell J.S., Todd A.J. (2010). Populations of inhibitory and excitatory interneurons in lamina ii of the adult rat spinal dorsal horn revealed by a combined electrophysiological and anatomical approach. Pain.

[45] Mercer Lindsay N., Chen C., Gilam G., Mackey S., Scherrer G. (2021). Brain circuits for pain and its treatment. Sci. Transl. Med..

[46] Kim S.K., Nabekura J. (2011). Rapid synaptic remodeling in the adult somatosensory cortex following peripheral nerve injury and its association with neuropathic pain. J. Neurosci..

[47] Takeda I., Yoshihara K., Cheung D.L., Kobayashi T., Agetsuma M., Tsuda M., Eto K., Koizumi S., Wake H., Moorhouse A.J. (2022). Controlled activation of cortical astrocytes modulates neuropathic pain-like behaviour. Nat. Commun..

[48] Yu J.M., Hu R., Mao Y., Tai Y., Qun S., Zhang Z., Chen D., Jin Y. (2023). Up-regulation of hcn2 channels in a thalamocortical circuit mediates allodynia in mice. Natl. Sci. Rev..

